# Genetic Aspects of Alcohol Use and Alcoholism in Women

**Published:** 1994

**Authors:** Dace S. Svikis, Martha L. Velez, Roy W. Pickens

**Affiliations:** Dace S. Svikis, Ph.D., is an assistant professor and Martha L. Velez, M.D., is a research associate at the Johns Hopkins University School of Medicine, Baltimore, Maryland. Roy W. Pickens, Ph.D., is a senior research scientist at the Intramural Research Program, National Institute on Drug Abuse, Rockville, Maryland

## Abstract

The factors influencing alcohol use and alcoholism are not the same in women as in men. Genetic factors may contribute to these differences.

Genetic studies are a crucial component of alcohol research. These studies attempt to quantify genetic influences on problem drinking; characterize patterns of inheritance; and, ultimately, identify the gene or genes that confer susceptibility to alcoholism. Results of such research have important implications for the prevention, early detection, and treatment of alcoholism.

There is reason to believe that the factors influencing alcohol use in general, and alcoholism in particular,^1^ are not the same in women as in men (National Institute on Alcohol Abuse and Alcoholism [NIAAA] 1993). Nevertheless, research on the genetics of alcoholism has traditionally used male subjects or has pooled the data in a manner that obscures gender differences.

Genetic factors may influence gender differences in alcohol use and alcoholism in two ways. First, the same genetic influences may exist in both women and men, with the observed gender differences being attributable entirely to environmental factors. Second, different (but possibly overlapping) genetic influences may be responsible for the observed gender differences. This article discusses available evidence for distinguishing between these two alternatives based on four types of studies: family, adoption, and twin studies; molecular genetic studies; biological marker studies; and animal studies.

## Family, Adoption, and Twin Studies

### Family Studies

Family studies examine rates of alcoholism in relatives of the alcoholic and nonalcoholic study subjects (probands). Such studies have consistently found the risk for alcoholism to be 4 to 7 times greater in relatives of alcoholics compared with relatives of nonalcoholics (Cotton 1979; Merikangas 1990). Overall, male relatives are more likely to be alcoholic than are female relatives.

Few studies have compared male alcoholic probands and female alcoholic probands with respect to the occurrence of alcoholism among their relatives. In such studies, gender differences are suggested by different rates of transmission of alcoholism to same-gender versus opposite-gender relatives. For example, if the female relatives of female probands have the same prevalence of alcoholism as do the female relatives of male probands, then no gender differences are indicated. However, if the female relatives of female probands have higher (or lower) rates of the disorder than do female relatives of male probands, then gender differences are suggested.

In a recent review of the literature, McGue and Slutske (1993) identified only seven family studies that examined alcoholism in female and male relatives (mostly parents) of both female and male alcoholic probands. In general, female and male relatives had similar rates of alcoholism regardless of whether the probands were male or female. Alcoholism was found in 6 percent of female relatives of male probands and 10 percent of female relatives of female probands. Alcoholism was found in 27 percent of male relatives of male probands and 33 percent of male relatives of female probands.

Thus, family studies have not found evidence for a different genetic influence on alcoholism in women compared with men. However, the possibility remains that gender-specific genetic influences do exist but are overshadowed by environmental influences.

### Adoption Studies

Adoption and twin studies provide more definitive evidence of genetic influences than do family studies. In adoption studies, alcoholism rates of adopted-away children of alcoholic parents are compared with alcoholism rates of adopted-away children of nonalcoholic parents. Genetic influences are indicated by higher alcoholism rates in the children of alcoholic than nonalcoholic parents.

Four adoption studies have examined the role of genetic factors in alcoholism in women. In the first study, Roe (1944) determined alcoholism rates in adopted daughters whose biological fathers were heavy drinkers. These rates were found to be not significantly different from alcoholism rates in the adopted-away daughters of nonalcoholic fathers. This same study also failed to find higher alcoholism rates for sons of alcoholic fathers.

Similarly, Goodwin and colleagues (1977) compared adopted-away daughters of alcoholic parents with a matched sample of adopted-away daughters of non-alcoholic parents. In 85 percent of the cases, the father was the alcoholic biological parent. The rates of alcoholism in adopted-away daughters of alcoholic and nonalcoholic parents were identical (4 percent), providing no support for the role of genetic factors in the development of female alcoholism. In contrast, another study by Goodwin and colleagues (1973) found evidence of genetic influences in male alcoholism—alcoholism rates were 18 percent in adopted-away sons of alcoholic parents and 5 percent in adopted-away sons of nonalcoholic parents.

In the third study, Bohman (1978) initially reported no evidence for genetic factors in adopted-away daughters of alcoholic and nonalcoholic biological parents. Subsequently, these investigators (Bohman et al. 1981) reanalyzed their data and compared the influence of paternal and maternal alcoholism on the development of alcoholism in the adoptees. If their biological mothers were alcoholic, female adoptees were more than three times more likely to be alcoholic (10 percent) than if their biological mothers were not alcoholic (3 percent). These results must be interpreted with caution because of the small sample size (51 alcoholic biological mothers).

In cases where the biological *father* of the adoptee was alcoholic, results varied with the severity of the father’s alcoholism. Daughters of fathers with mild alcoholism showed increased rates of alcoholism. Daughters of fathers with severe alcoholism showed no increased rate of alcoholism. In this same population sample, Cloninger and coworkers (1981) identified 862 adopted sons. Rates of alcoholism were significantly higher in sons with an alcoholic biological parent of either gender (22 to 28 percent) than in those with a nonalcoholic biological parent (15 percent). Greater genetic effects were seen in male adoptees with moderate than with mild or severe alcoholism.

Results of the fourth study (Cadoret et al. 1985) strongly supported the role of genetic factors in the development of alcoholism in women. Specifically, 33 percent of adopted-away daughters with at least one alcoholic biological parent met standard psychiatric criteria for alcohol abuse or dependence, compared with only 5 percent of adopted-away daughters with no alcoholic biological parents. Findings were similar for sons: alcoholism rates were 62 percent in adoptees with an alcoholic biological parent and 24 percent in adoptees with neither parent alcoholic.

When data from the four studies are combined, the rate of alcohol abuse in adopted-away daughters of alcoholics (4.7 percent) is only slightly higher than the rate of alcohol abuse in adopted-away daughters of nonalcoholics (3.1 percent), suggesting a minimal role for genetic factors in female alcoholism (McGue and Slutske 1993). It is difficult to draw firm conclusions from these data, however, because of the modest sample sizes involved: only 41 female adoptees with alcohol problems were included in all of the studies (compared with 202 alcohol-abusing male adoptees).

### Twin Studies

An alternative strategy for evaluating genetic and environmental influences is the twin study. This type of study is based on the existence of two types of twins: identical (monozygotic, or MZ), and fraternal (dizygotic, or DZ). MZ twins share 100 percent of their genes, whereas DZ twins are like ordinary siblings, sharing on average only 50 percent of their genes. A genetic influence is indicated by greater correlation (concordance) of alcoholism with MZ than with DZ twins.^2^

#### Genetic Influence on Alcohol Consumption

Studies of men have found higher concordances for MZ than for DZ twins with respect to quantity and frequency of drinking. Heritabilities^3^ ranged from 0.16 to 0.82 (Partanen et al. 1966; Jonsson and Nilsson 1968; Kaprio et al. 1987). Swan and coworkers (1990) recently reported a heritability for alcohol use (i.e., non-abstention) of 0.43, even after other factors that might have contributed to concordance rate differences were controlled (e.g., coffee consumption, contact between the twins, and certain personality traits). Pedersen (1981) reported genetic influences in quantity and frequency of alcohol consumption in both genders. More recently, Heath and colleagues (1991*a*,*b*) reported a twin study of alcohol consumption in approximately 4,000 male and female twins. Genetic factors were found to contribute to frequency of alcohol use in both women and men. Heritabilities were estimated to be 0.66 in women and either 0.42 or 0.75 (depending on the model employed) in men. Similarly, genetic influences were found to contribute to quantity of use in both genders, with heritability estimates of 0.57 in women and either 0.24 or 0.61 in men. Clifford and coworkers (1984) found evidence of genetic influences in alcohol use by women but not in men.

#### Genetic Influence on Alcoholism

Whereas twin studies have consistently found evidence of genetic influences in alcohol *use* by women, the results of twin studies of *alcoholism* in women have been less consistent. In the four twin studies of female alcoholism where heritability estimates could be calculated, values ranged from 0.00 (McGue et al. 1992) and 0.10 (Caldwell and Gottesman 1991) to 0.42 (Pickens et al. 1991) and 0.56 (Kendler et al. 1992). The highest heritability estimate was found in the largest study (i.e., Kendler et al. 1992), which was the only study in which subjects were ascertained from the general population. In contrast, twin studies of alcoholism in men have yielded more consistent findings, with four of the five studies supporting a role for genetic factors. This inconsistency in results may in part reflect differences in methodology among the studies. For example, subjects in Kendler’s study were selected from a twin registry, whereas the other studies obtained subjects from alcoholism treatment centers. The latter procedure may introduce a bias into the interpretation of data. Heritability estimates for alcoholism in men have consistently ranged from 0.50 to 0.60 (McGue 1994), including data from both population-based and clinically based studies.

## Molecular Genetic Studies

The studies discussed above were designed to quantify genetic influences and characterize patterns of inheritance. Molecular genetic studies attempt to identify specific genes or gene markers that are associated with alcoholism. To date, research has failed to identify a genetic marker for alcoholism in either women or men (Merikangas 1990).

Several researchers have reported an association between alcoholism and a specific variant of the gene for the D2 dopamine receptor. This receptor, a component of certain brain cells, appears to be involved in the addictive effect of alcohol and other drugs (Wise 1988). Although several studies have reported a strong association between the variant gene and alcoholism (e.g., Blum et al. 1990; Comings et al. 1991), others have failed to replicate these findings (e.g., Bolos et al. 1990; Parsian et al. 1991). Methodological issues, such as inconsistent diagnostic criteria for alcoholism and failure to apply appropriate controls, may account for some of these differences.

Although both men and women have been included in a number of the D2 dopamine receptor gene studies, most studies failed to report the data separately for men and women. In addition, as in the twin and adoption studies summarized above, the actual number of females tested in the association studies published to date has been small (McGue and Slutske 1993). For these reasons, it is not possible to determine whether gender differences are present in the association of the variant gene and alcoholism.

## Biological Marker Studies

Biochemical traits, or markers, that are inherited along with alcoholism can help identify persons at high risk. Examples of such markers include brain chemicals and enzymes involved in alcohol metabolism. No biological marker studied to date is highly predictive of the development of alcoholism. In addition, no studies have found gender to be a factor in the association between a biological marker and alcoholism risk. Studies to determine if alcoholism is linked to the female sex (X) chromosome also have yielded negative results (e.g., Kaij and Dock 1975).

One strategy for investigating biological factors in alcoholism is the high-risk study. In this design, subjects at increased risk for developing alcohol problems are compared with subjects not at increased risk. A family history of alcoholism is often used to characterize individuals as either high risk (having one or more alcoholic first-degree relatives^4^) or low risk (having no alcoholic first-degree relatives). The high-risk group is typically labeled family history positive (FHP), whereas the low-risk group is labeled family history negative (FHN).

Many high-risk studies compare responses of FHP subjects with responses of FHN subjects following administration of standard doses of alcohol (alcohol challenge). The majority of studies to date have focused exclusively on sons of alcoholics. The few studies conducted with women have revealed several differences between FHP and FHN subjects. One study measured subjects’ ability to maintain a stable upright posture after alcohol consumption. FHP women exhibited less body sway than did FHN women at a peak blood alcohol concentration of 0.08 percent, the legal criterion of intoxication in many States (Lex et al. 1988*a*). FHP women also made fewer errors than did FHN women on a test of visual information processing administered 30 minutes after alcohol ingestion (Lex et al. 1988*b*; Lex et al. 1994). Alcohol’s ability to reduce stress was more marked in FHP women than in FHN women, as measured by changes in heart rate and other responses (Levenson et al. 1987).

One area that has received some attention in women is a component of brain electrical activity called P300. This “brain wave” is associated with general cognitive functioning and the ability to identify and interpret subtle stimuli in the environment (Schuckit 1992). The amplitude (height) of the P300 brain wave, as measured with an electroencephalogram, has been suggested as a biological marker for alcoholism in men (Begleiter and Porjesz 1988). Alcoholic women have decreased P300 amplitudes compared with both their nonalcoholic sisters and FHN controls (Hill and Steinhauer 1993*a*). Although prepubertal FHP males showed a lower amplitude of P300 than prepubertal FHN males, no difference between FHP and FHN prepubertal females was found (Hill and Steinhauer 1993*b*).

## Animal Studies

Animal studies offer another approach for examining the role of genetic factors in alcoholism and the mechanisms by which genes exert their influence. Most experimental animals used for this purpose are either inbred strains or selectively bred lines^5^ (Crabbe 1989). Inbred strains are created by pairing close genetic relatives until they can be considered genetically identical. The majority of alcohol studies using inbred strains have employed only male animals or else they fail to differentiate between males and females in the analysis of the data (e.g., Suzuki et al. 1988).

Selective breeding is used to develop animal lines that differ in their genetic sensitivity or responsivity to specific effects of alcohol. The ability to breed animals for a particular trait proves that genetic factors play a role in the development of that trait (Phillips et al. 1989). Mouse and rat lines are currently available for numerous alcohol-related characteristics.

As with inbred strains, the majority of selective breeding studies have failed to differentiate between males and females in the data analysis. Li and colleagues (1993) reviewed the alcohol-preferring (P) and alcohol-nonpreferring (NP) rat lines, presenting daily mean alcohol consumption separately for male and female P and NP rats. The data suggest the possibility of gender differences, particularly in the P line, with male rats showing mean consumption of 5.3 to 5.7 g/kg/day and female rats averaging 6.5 to 7.3 g/kg/day. The investigators do not report statistical analyses of these data, but the values highlight the need for research examining gender differences in selectively bred mice and rats.

## Summary

Epidemiological and clinical data suggest significant differences in both alcohol use patterns and alcoholism between women and men (NIAAA 1993). This article reviewed research examining the extent to which genetic factors may contribute to these gender differences. In studies of alcohol use, the most compelling evidence comes from twin studies involving large population-based samples. Findings suggest genetic factors play a small-to-moderate role in determining quantity and frequency of alcohol consumption in both male and female twins.

For the clinical disorder of alcoholism, fewer studies with smaller sample sizes preclude definitive conclusions. However, when data from multiple studies are combined, family studies suggest familial influences are similar in men and women, although these findings do not negate the possibility of genetic differences. For women, adoption and twin studies have produced mixed results, with half of the studies reporting no genetic effects for women and the other half supporting a role for genetic factors in female alcoholism. The only twin study to select subjects from a twin registry produced the highest estimates of heritability (Kendler et al. 1992). In contrast, the data for males are more definitive, with the majority of adoption and twin studies supporting a genetic contribution to male alcoholism.

With other strategies (e.g., molecular genetic studies), either women have not been included as subjects or the investigators have failed to examine the data separately by gender. Whereas gender differences are evident in the response of FHP and FHN subjects to alcohol challenge, results may be questioned because of the small number of female subjects in most studies. In the animal research area, virtually no attention has been paid to gender differences.

More studies involving larger numbers of women as subjects are needed to establish the role of genetic factors in female alcoholism.

## Footnotes

1 In this article, the term “alcoholism” indicates alcohol dependence or addiction, whereas “alcohol abuse” and “heavy drinking” refer to nonaddictive problem drinking. The exact definitions and diagnostic criteria for these terms vary widely, however, depending upon the reference cited. Similarly, severity of alcoholism is defined differently by different researchers. The term “alcohol problems” refers to drinking patterns that have caused negative social, personal, or medical consequences.

2 Concordance indicates the extent to which a trait is found in both members of a twin pair. A concordance rate of 0.5 indicates that when one twin expresses the trait, the other twin expresses the trait in 50 percent of the cases.

3 Heritability is a statistical estimate of the proportional contribution of genetic influence to an observed trait.

4 First-degree relatives include a person’s parents, offspring, and siblings.

5 Other animal models exist; see Crabbe 1989 for review.
